# The Differential Associations Between Passive and Interactive Screentime and Sleep Duration Among 8th and 11th Grade Adolescents

**DOI:** 10.3390/children13010127

**Published:** 2026-01-15

**Authors:** Christopher D. Pfledderer, Nalini Ranjit, Debra Saxton, Adriana Pérez, Deanna M. Hoelscher, Natalie P. Archer

**Affiliations:** 1 Department of Health Promotion and Behavioral Sciences, School of Public Health in Austin, University of Texas Health Science Center at Houston (UTHealth), 1616 Guadalupe, Suite 6.340, Austin, TX 78701, USA; 2 Michael & Susan Dell Center for Healthy Living, School of Public Health in Austin, University of Texas Health Science Center at Houston (UTHealth), 1616 Guadalupe, Suite 6.300, Austin, TX 78701, USA; 3 Maternal & Child Health Unit, Texas Department of State Health Services, P.O. Box 149347, Austin, TX 78714, USA; 4 Department of Biostatistics and Data Science, School of Public Health in Austin, University of Texas Health Science Center at Houston (UTHealth), 1616 Guadalupe, Suite 6.340, Austin, TX 78701, USA; 5 Cancer Epidemiology and Surveillance Branch, Texas Department of State Health Services, P.O. Box 149347, Austin, TX 78714, USA

**Keywords:** screentime, sleep, adolescents

## Abstract

**Highlights:**

**What are the main findings?**

• Watching TV and playing video/computer games has differential associations with sleep duration among adolescents, and these associations differ by grade, gender, and ethnicity.

**What are the implications of the main findings?**

• These contextual differences in screentime should be considered when exploring how media use influences other health behaviors and outcomes. Researchers and policymakers involved in creating screentime guidelines should take into account the differences between passive and interactive media use. In essence, not all screentime is created equally.

**Abstract:**

**Background:** Although several studies have reported associations between screentime and shortened sleep duration among adolescents, contextual relationships between different forms of screentime are not well understood. The purpose of this study was to examine how television (TV) watching (passive media use) and video/computer gaming (interactive media use) are associated with short sleep duration among 8th and 11th grade adolescents. **Methods:** We used data from adolescents (8th and 11th grade students) who participated in the Texas School Physical Activity and Nutrition (Texas SPAN) survey in 2015–2016. Sleep duration was the outcome variable, which was dichotomized into short sleep duration (less than 8 h) and meeting sleep recommendations (more than 8 h). Independent variables included daily TV screentime and video/computer game screentime. We used weighted logistic regression models to understand associations between sleep duration and both TV screentime and video/computer game screentime. **Results:** Among both 8th grade boys and Hispanic 8th grade girls, spending more than 2 h/day playing video/computer games was associated with greater odds of shorter sleep duration. Among 11th graders, TV screentime was associated with lower odds of shorter sleep duration. **Conclusions:** Watching TV and playing video/computer games have differential associations with sleep duration among adolescents, and these associations differ by grade, gender, and ethnicity. Researchers and public health agencies interested in associations between meeting sleep recommendations and screentime in adolescents should consider these contextual differences when designing and conducting studies related to electronic media use and sleep.

## 1. Introduction

Adolescence is a time of significant physical, emotional, and intellectual maturation, and sleep is necessary for adolescents’ development, growth, and quality of life. Chronic sleep loss in adolescents has been linked to increased risk for several adverse health outcomes and behaviors, including mood disorders, obesity, injuries, and alcohol and drug use [1,2]. Research has found that adolescents often get inadequate amounts of sleep on a regular basis, and the prevalence of adequate sleep has decreased among high school students in the United States [3,4]. Although sleep duration tends to decline as students get older, prevalence of insufficient sleep is high among both younger and older adolescents, with similar trends seen into adulthood [5,5,6]. Many factors have been shown to contribute to inadequate sleep in adolescents, including the influence of biological processes, electronic media use and screentime, school start times, caffeine, total adiposity, and feeling unsafe in one’s neighborhood [2,6,7,8,9,10,11,12,13].

Recent research has shown evidence of deleterious associations between screentime and sleep among youth [14]. Several theoretical models have been proposed by which electronic media use might be shown to impact quality and quantity of sleep. It has been suggested that electronics may affect sleep by simply displacing sleep time, thus shortening sleep duration [15,16]. Use of electronic media may displace sports activities, making sleep more difficult [17]. Additionally, electronic media use may cause poor-quality sleep through excessive violent or stimulating themes, as is offered in various computer and video games [18]. Electronic media use may also inhibit sleep through bright light exposure inherent in TV and computer screens, suppressing melatonin and delaying the circadian rhythm [16,19,20]. Further, electronic media could impair sleep due to physical discomfort, such as muscular pain and headache, which can be caused by extended electronic media use, such as computer gaming [16]. Understanding which specific types of media use most strongly influence short sleep duration among adolescents is critical for developing targeted interventions and refining current evidence-based recommendations for screentime.

Most studies examining associations between screentime and sleep among adolescents have either looked at screentime exposure to only one type of electronic media (e.g., TV, computers, video games, or mobile devices), or have combined multiple types of media into one collective measure of media use [16,19]. This approach has inherent weaknesses as it obscures potentially important differences in how various media types influence sleep and subsequently limits the specificity of public health recommendations for screentime exposure. However, there is increasing evidence that perhaps not all types of screentime influence sleep equally. Particularly, some studies have suggested that interactive media use (such as cell phone use and computer/video gaming) may be more disruptive to sleep than passive media use (such as TV watching) [16]. Factors such as heightened cognitive engagement and arousal which can come with interactive media use may intensify the sleep-disrupting mechanisms described above, yet few studies have tested whether these differential associations exist. In short, relatively few studies have examined whether associations between screentime and sleep differ across different types of electronic media use, and even fewer have assessed how associations may differ among adolescents of different ages [20,21]. Without age-specific evidence comparing media types, recommendations for screentime usage remain generalized and may miss opportunities to target more detrimental screentime behaviors for different age groups.

The purpose of this study was to determine whether and how two separate types of screentime (TV [passive] and computer/video games [active]) are associated with sleep duration among 8th and 11th grade adolescents in a statewide representative sample in Texas, using the 2015–2016 Texas School Physical Activity and Nutrition Survey (Texas SPAN). Research questions included the following: (1) Are TV screentime and time spent video/computer gaming associated with sleep duration among 8th and 11th graders in Texas? (2) Are TV screentime and time spent video/computer gaming differentially associated with sleep duration among 8th and 11th graders in Texas?

This study has the potential to make unique contributions to the literature. First, by examining passive and interactive media separately within the same population, we can directly compare their relative associations with sleep duration, providing clearer guidance for intervention priorities. Second, by analyzing these associations separately for 8th and 11th grade students, we may be able to provide developmentally specific evidence that can inform age-appropriate recommendations. These contributions address critical gaps in the current literature and advance our understanding of modifiable factors contributing to insufficient sleep among adolescents.

## 2. Materials and Methods

### 2.1. Study Design and Data Collection

We conducted secondary data analyses of the Texas School Physical Activity and Nutrition (SPAN) 2015–2016 survey for 8th and 11th grade students. Texas SPAN is a cross-sectional statewide survey designed to determine the prevalence of school-aged child and adolescent overweight and obesity in Texas, as well as related diet and physical activity behaviors. Texas SPAN uses a stratified, multistage sampling plan to obtain state-representative data for Texas school-aged children and adolescents [22]. Further details of the Texas SPAN survey and its sampling scheme have been described elsewhere [23]. Trained field staff administered Texas SPAN survey questionnaires to students at randomly selected public schools throughout Texas. Survey questions asked about their demographic information, diet, nutrition, physical activity, and other behaviors that could be associated with overweight and obesity including sleep, which were all self-reported by students. Approval for the Texas SPAN study was obtained from the Committee for the Protection of Human Subjects at the University of Texas Health Science Center at Houston (UTHealth) (#HSC-SPH-00-056) and the Texas Department of State Health Services (DSHS) Institutional Review Board (#04-062). Participating school districts and schools also reviewed study protocols for compliance with school human subjects and research regulations.

### 2.2. Measures

To measure sleep duration, students were asked, “On an average school night how many hours of sleep do you get?” The response options were as follows: 4 or fewer hours, 5 h, 6 h, 7 h, 8 h, 9 h, and 10 or more hours. The outcome variable in this study was sleep duration, dichotomized as less than eight hours of sleep on a school night (defined as short sleep duration) or eight or more hours on a school night (recommended sleep duration), per the minimum American Academy of Sleep Medicine sleep recommendations for adolescents aged 13 to 18 years [24].

Exposure variables of interest were reported use (outside school) of two different types of electronic media: TV and video/computer games. TV screentime was operationalized with the question, “How many hours per day do you usually watch TV, DVDs, or movies away from school for anything except schoolwork? Count internet surfing, instant messaging, or chatting. Do not count schoolwork, games.” Video/computer gaming time was operationalized with the question, “How many hours per day do you usually spend playing video or computer games away from school? Count games on your video game consoles (Nintendo^®^, Xbox^®^, Playstation^®^), computer or handheld (e.g., Minecraft^®^, Madden NFL^®^, Pokemon^®^), and games on your phone or mobile device (e.g., Candy Crush^®^, Angry Birds^®^).” Responses for both questions used a numeric scale from less than one hour to eight or more hours, and also included an ‘I don’t watch TV’/‘I don’t play video or computer games’ option. For this study, responses were collapsed into three categories: no TV (or no video/computer games), less than 2 h daily (but more than none), and 2 or more hours daily. These categories are consistent with the American Academy of Pediatrics’ (AAP’s) 2013 policy statement that was in effect when survey data were collected, and this cutoff has been used in several other studies of electronic media use and sleep [2,16].

Demographic data included student gender and Hispanic ethnicity. Other categorical measures considered for inclusion in the analyses, because of potential associations with sleep duration, were as follows: (1) student body mass index (BMI) for age weight status categories (underweight, normal or healthy weight, overweight, or obese) [25]; (2) highest level of parental educational attainment (less than high school, high school or GED, or more than high school); (3) a composite categorical measure of school safety based on responses to 5 separate survey questions regarding how safe a student felt in various locations at school including the classroom, school grounds, cafeteria, going to and from school, and in the restroom, with responses to each of these questions being ‘scared and unsafe’, ‘kind of safe’, and ‘very safe’; and (4) whether or not a student met the recommended daily physical activity levels (≥60 min moderate to vigorous physical activity) set by the US Department of Health and Human Services (yes or no).

### 2.3. Statistical Analysis

Descriptive analyses included weighted proportions and 95% confidence intervals for each of the measures listed above, stratified by grade. For each measure, weighted proportions were compared between grade levels using a weighted Rao–Scott Chi-square test. To ensure that there was not a high degree of multicollinearity between our two electronic media types of interest, TV screentime and video/computer gaming, the degree of correlation between these two variables was assessed using Spearman’s rank correlation coefficient test. Two weighted multiple logistic regression analyses were conducted to determine associations between each type of electronic media use and short sleep duration for each grade, separately. Sampling weights with Taylor linearization methods were used in analyses to take into account the complex design of the Texas SPAN data. All analyses were conducted using SAS, version 9.4.

**Figure 1:**
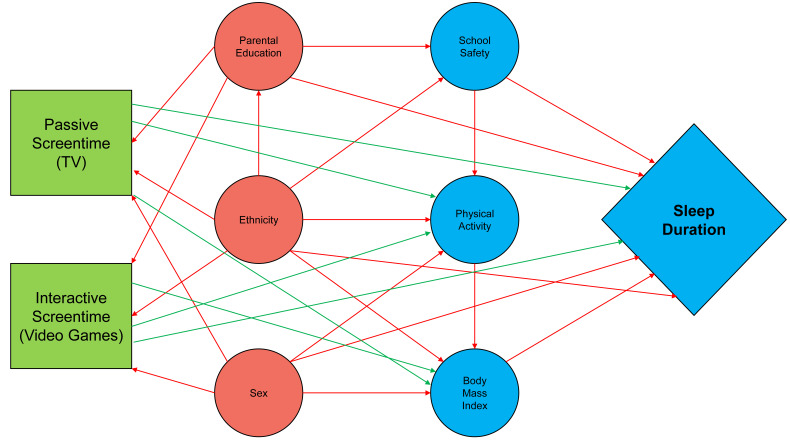
Directed acyclic graph (DAG) including predictors of interest, potential confounding variables, and outcomes (sleep duration) for each grade level.

We used a directed acyclic graph (DAG) to identify a minimum set of confounding variables to account for in multiple logistic regression analyses (Figure 1). Relationships between the variables shown were identified based on associations found in the previous literature [26,27,28,29,30,31], and minimum confounding variables that we needed to control for are indicated in red on the graph. Based on these results, parental educational attainment, student ethnicity, and student gender were included in all weighted logistic regression models, either as covariates or as stratifying variables (Figure 1). BMI and physical activity were not included because these had the potential to mediate the association between either type of screentime and sleep duration. Both types of electronic media use were included in each model, so that any associations between TV screentime and sleep duration were adjusted simultaneously. The DAG was constructed using the ‘daggity’ package in R (Version 4.1.3).

Because we sought to evaluate the effect of electronic media use on sleep duration by gender or ethnicity, four interaction terms (gender*TV screentime, gender*video/computer gaming duration, ethnicity*TV screentime, and ethnicity*video/computer gaming duration) were added to the weighted multiple logistic regression models for each grade. Only statistically significant interaction terms (*p* ≤ 0.05) were included in the final models. Non-significant interaction terms were removed using manual backwards elimination. If the interaction term was significant, we further stratified the analyses as needed (by gender, ethnicity, or both). Tests of multicollinearity revealed a mean variance inflation factor of 1.89 (range = 1.08–3.94). The type I error level used to declare statistical significance was 0.05.

## 3. Results

### 3.1. Demographics

During 2015–2016, 5421 8th grade students and 3635 11th grade students were surveyed. Of these, 1154 surveys were excluded from analysis because of missing outcome or predictor variable data. Specifically, 319 (3.5%) participants did not have sleep data, 150 (1.7%) participants did not have data for computer/video game usage, and 140 (1.5%) participants did not have data for TV watching time. An additional 545 (6.0%) participants had missing data for covariates. The final sample included 4520 8th grade students (mean age = 13.6 years, range = 11–16 years) and 3382 11th grade students (mean age = 16.5 years, range = 14–17 years), representing a total of 584,078 adolescents in Texas. Table 1 displays participant demographics, sleep duration characteristics, and electronic media use behaviors by grade level. Overall, a significantly lower proportion of 8th graders reported sleeping less than 8 h on school nights compared with 11th graders (48.7% vs. 71.0%; *p* < 0.0001). A higher proportion of eighth graders reported playing video/computer games daily compared with eleventh graders (*p* < 0.001). No statistically significant differences in gender, ethnicity, parental educational attainment, or daily TV screentime were observed between the two grades. Among both 8th and 11th graders, TV screentime and video/computer gaming were only weakly correlated with each other (r = 0.2 for both grades).

### 3.2. Total Sample Logistic Regression Results

Weighted logistic regressions results for the total sample (8th and 11th graders together) revealed a statistically significant and negative association between video/computer game usage and sleep duration (Supplementary Table S1) for those who played video/computer games for more than 2 h (OR = 0.7; 95% CI = 0.6–0.8). Further, a statistically significant and positive association between TV screentime and sleep duration was found for both those who watched TV for less than two hours (OR = 1.4; 95% CI = 1.1–1.7) and those who watched TV for more than two hours (OR = 1.3; 95% CI = 1.1–1.6) compared to those who watched no TV.

### 3.3. Interactions

Weighted logistic regression results for both 8th and 11th grade students overall showed a significant interaction between student gender and daily video/computer gaming duration (*p* = 0.02 for 8th graders, *p* = 0.01 for 11th graders). In addition, among 8th grade girls, a significant interaction between Hispanic vs. non-Hispanic ethnicity and TV screentime was observed (*p* = 0.002).

### 3.4. Eighth Grade Students

Table 2 presents weighted logistic regression results for 8th graders. Daily TV screentime was not statistically significantly associated with sleep duration among 8th grade boys (OR = 0.9; 95% CI = 0.4–2.0) after adjusting for Hispanic ethnicity, parental education, and video/computer gaming time. Eighth grade boys who spent less than 2 h/day playing video or computer games had 2.8 times the odds of a short sleep duration when compared with boys who did not play video/computer games (95% CI = 1.4–5.7), and those who spent 2 or more hours/day playing video/computer games had 3.6 times the odds of a short sleep duration (95% CI = 1.6–8.1) after controlling for Hispanic ethnicity, parental education, and TV screentime. Hispanic 8th grade girls who spent 2 or more hours playing computer or video games also had significantly increased odds of short sleep duration (OR = 2.3; 95% CI = 1.1–4.7) compared to those who did not play computer or video games after adjusting for parental education and TV screentime, but this association was not observed among non-Hispanic 8th grade girls. Hispanic 8th grade girls who watched TV for two or more hours daily had significantly lower odds of short sleep duration (OR = 0.4; 95% CI = 0.2–0.8) compared with Hispanic 8th grade girls who did not watch TV after adjusting for parental education and video/computer gaming time.

### 3.5. Eleventh Grade Students

Table 3 presents weighted logistic regression results for 11th graders. Among 11th graders, boys who watched any amount of TV daily had significantly lower odds of a short sleep duration (OR = 0.2; 95% CI = 0.1–0.5) compared with boys who did not watch TV after adjusting for Hispanic ethnicity, parental education, and video/computer gaming time. Eleventh grade girls who watched 2 or more hours of TV daily also had lower odds of a short sleep duration (OR = 0.2; 95% CI = 0.1–0.6) compared with girls who did not watch TV after controlling for Hispanic ethnicity, parental education, and video/computer gaming time. A significant interaction between Hispanic ethnicity and video/computer gaming time (*p* = 0.02) was observed among 11th grade girls; however, models with and without this interaction term resulted in similar estimates of the association between video/computer gaming time and sleep duration, suggesting that the magnitude of the interaction effect was small. In the interest of parsimony, we opted to analyze all 11th grade girls together as a single model. Video/computer gaming duration was not significantly associated with short sleep duration among either 11th grade boys or girls.

## 4. Discussion

Study results indicated that passive (TV watching) and interactive (video/computer gaming) electronic media use each had differential associations with sleep duration for this statewide representative sample of 8th and 11th grade students in Texas. These effects also differed by grade, gender, and ethnicity. Results suggest that not all screentime is created equal with regard to its influence on sleep duration, even though current sleep recommendations and most studies treat screentime/electronic media use as a single or compositive variable, encompassing a wide range of electronic media types [24].

Spending time playing video/computer games was associated with increased risk of a short sleep duration among younger (8th grade) adolescents, specifically among 8th grade boys of all ethnicities and Hispanic girls. However, video/computer game duration did not influence sleep duration among older (11th grade) adolescents. This differential association found between 8th and 11th graders may reflect important development transitions in self-regulation and time management skills between 8th and 11th graders. Executive function, including inhibitory control and working memory, continues to develop throughout adolescence, with significant maturation occurring between middle and high school [32]. As adolescents progress through high school, they may develop more sophisticated strategies for balancing competing demands on their time, including gaming, homework, and sleep [33]. Additionally, older adolescents may have greater autonomy in setting their own schedules and may have learned through experience the consequences of inadequate sleep on their academic performance and daily functioning [34]. The specific vulnerability of 8th grade boys and Hispanic girls to gaming-related sleep loss warrants particular attention, as these groups may benefit most from targeted interventions. While several other studies that examined adolescent computer or video gaming have also found associations with short sleep duration [15,35], there have been relatively few studies to date that have examined the relationship between this specific type of electronic media use (computer/video gaming) and sleep duration [16,36].

Bruni et al. [13] found that late evening screentime activities for older adolescents (ages 14–16) more frequently consisted of Internet or mobile phone use, whereas video gaming was more frequently seen among preadolescents (ages 11–13 years). Further, when analyzing sleep onset latency (SOL), a study with older adolescents found that video gaming had little effect on SOL [37], whereas another study with younger adolescents observed a stronger association between gaming time and SOL [38]. It is possible that among older adolescents, there may be an attenuated association between video/computer gaming and other sleep outcomes as well, including sleep duration [39]. The shift from video gaming to other forms of interactive media as adolescents age may explain why we observed no significant association between gaming and sleep among 11th graders. This developmental shift in media preferences suggests that interventions should be age-tailored, focusing on video gaming for middle school students while addressing social media and smartphone use for high school students, which is something that was not performed in this study.

Surprisingly, daily TV screentime was not negatively associated with sleep duration among 11th grade adolescents (both boys and girls). Those who watched at least some TV each day were more likely to obtain a sufficient amount of sleep than those who watched no TV. For 8th graders, this protective association was also observed among Hispanic girls. Findings from the literature relating to this association are mixed. While multiple studies on TV viewing and adolescent sleep have reported associations between increased TV watching and shorter sleep duration [20,21,35,40], there have also been several studies that found no significant association between TV screentime and sleep duration [30,41,42]. At least one study reported a protective association [43], as we observed in this study. Our results are also consistent with previous findings that passively watching TV is likely less disruptive to sleep than the use of more interactive electronic media, such as video games or cell phones [44,45,46]. The lack of interpersonal interaction and the lower cognitive engagement required for TV viewing may result in less psychological and physiological arousal compared to interactive media [37,47,48]. The interactive nature of video games has been consistently shown to increase physiological arousal, with elevated heart rate and reduced subjective sleepiness [47,48], making it easier for adolescents to subsequently fall asleep after passive TV viewing. Recent evidence confirms that interactive screentime like gaming is more strongly associated with sleep disruption than passive media consumption [48].

Only a small proportion of adolescents (7–8%) reported watching no TV. It is possible that adolescents who watch no TV may differ from those who watch at least some TV daily in a variety of ways which could impact sleep duration. For example, perhaps these adolescents exhibit higher engagement in extracurricular activities or spend more time on homework outside school. These busier schedules could contribute to shorter sleep durations compared to adolescents who watch at least some TV [12,49]. To explore this, we conducted additional analyses using only two categories of TV screentime duration: 2+ h/day vs. <2 h/day (including no TV watching). When using this new combined reference category, a difference in odds of short sleep duration was no longer observed among 8th grade girls who watched 2+ h of TV/day compared to those who watched <2 h of TV/day, regardless of ethnicity. Although lower ORs were still seen for both 11th grade boys and girls who watched 2+ h of TV/day, only 11th grade girls continued to have statistically significantly lower odds of a short sleep duration compared with those who watched <2 h of TV/day. These findings may be partially explained by the unmeasured characteristics of adolescents who watched no TV at all, rather than supporting the conclusion that TV viewing itself is associated with longer sleep duration.

The ethnicity- and gender-specific patterns we observed merit careful interpretation. The particular vulnerability of Hispanic girls in 8th grade to gaming-related sleep loss may reflect cultural norms regarding technology use, family structure, or other social factors that differentially influence media consumption patterns and sleep behaviors [50,51]. Gender differences across both grades suggest that boys and girls may use media differently, not just in terms of duration, but in the contexts and times of day they engage with different media types [52,53]. These disparities underscore the importance of culturally responsive and gender-sensitive approaches to promoting healthy sleep and media habits among adolescents.

The differential associations between media types and sleep duration have important practical implications for adolescent health and well-being. Inadequate sleep during adolescence has cascading effects on academic performance, with sleep-deprived students showing reduced attention, memory consolidation, and executive functioning, all critical for learning and school success [54,55,56]. Sleep restriction has been shown to impair working memory, sustained attention, and processing speed, observable even after partial sleep deprivation [57]. Indeed, studies have demonstrated that insufficient sleep is associated with poorer performance in cognitively demanding subjects such as mathematics and science, which require strong logical reasoning and problem-solving abilities [58,59]. Beyond academics, chronic sleep loss can impair mood regulation, may increase risk of depression and anxiety, and can compromise physical health through effects on immune function and metabolism [60,61]. Meta-analytic evidence shows that shorter sleep duration significantly increases the odds of mood deficits in adolescents, with particularly strong effects on positive affect, followed by depression, anger, and anxiety [62]. The relationship between sleep problems and mental health appears bidirectional, with sleep disturbances both predicting and resulting from anxiety and depressive symptoms [63]. For younger adolescents particularly, the interactive nature of video gaming may disrupt not just sleep quantity but also sleep routines and bedtime consistency, which are important for establishing healthy sleep hygiene patterns that persist into adulthood.

Our findings suggest several concrete public health recommendations for families, schools, and clinicians. First, parents and caregivers should prioritize limiting interactive media use for middle school students, as this appears to pose a risk to adequate sleep. Second, schools might consider incorporating age-appropriate education about the differential effects of media types on sleep into health curricula, empowering students to make informed choices about their media consumption. Third, pediatricians and school personnel working with adolescents experiencing sleep difficulties should specifically assess interactive screentime habits among younger teens and consider this a potential modifiable risk factor. Finally, public health messaging about screentime and sleep should move beyond generic recommendations to specify that interactive media use may pose particular risks for short sleep duration, especially for younger adolescents.

### Strengths and Limitations

Our study offers several strengths. Because of Texas SPAN’s statewide stratified, multistage sampling design, results should be generalizable to all 8th and 11th grade adolescents in Texas. The relatively large number of Texas SPAN participants allowed us to evaluate associations between electronic media use and sleep duration by grade and gender. Further, approximately 50% of all 8th and 11th grade SPAN respondents were Hispanic. This provided us with a large enough sample size to be able to adequately determine whether Hispanic ethnicity modified potential effects of screentime on sleep duration. Despite these strengths, our study also has limitations that should be acknowledged while interpreting findings.

Our results are based on self-reported data and do not include objective or physiological data on sleep. Many studies comparing self-reported sleep duration with objectively measured amounts of sleep (i.e., actigraphy) suggest that self-reports of average hours slept per night are often overestimated, indicating that problems with chronic sleep loss in adolescents may be even greater than the data indicate [2]. Because of the secondary nature of our analyses, our study used a limited number of types of media which were included in the survey, and did not include more recent forms of electronics, such as cell phones. We also were not able to examine electronic media multi-tasking among adolescents. There could have been other covariates of significance not measured by SPAN that might have been associated with both screentime and sleep duration, such as the timing of screen use each day or the amount of homework per night; however, we were unable to adjust for these in our model. Finally, regarding the secondary nature of our analyses, we were not able to treat sleep or screentime as continuous variables, which may have led to loss of information and reduced statistical power. Also, this study solely looked at sleep duration, not at sleep quality or any other sleep-related measures. Regarding sleep duration, a small number of participants in the sample categorized as meeting sleep recommendations (≥8 h sleep/night) may have exceeded the sleep recommendations (>10 h of sleep/night), which has been shown to be associated with negative health outcomes [24]. Future studies should explore how screentime context might influence health outcomes among those who exceed sleep recommendations as well. Finally, because the survey data used were cross-sectional, the temporality of the association between electronic media use and sleep duration could not be assessed.

## 5. Conclusions

This study highlights differences in associations between two types of electronic media use (watching TV and playing video/computer games) and sleep duration. Our findings suggest that passive electronic media use such as watching TV may not adversely influence sleep duration. However, active media use could contribute to short sleep duration, particularly among younger adolescents. Additionally, we found that the type of media used may impact adolescent sleep differently depending on age and gender. This information should be useful in helping to set public policies and recommendations regarding adolescent media use and when counseling adolescents and their families in a clinical setting.

## Figures and Tables

**Table 1:** Participant demographic, sleep duration, and electronic media use characteristics by grade level from Texas SPAN, 2015–2016.

Characteristic	8th Graders	11th Graders
	Unweighted n, Weighted % (95% CI)	Unweighted n, Weighted % (95% CI)
*Unweighted total (n)*	4520	3382
*Weighted total (N)*	294,373	289,705
*Sex*		
Boy	2305, 51.0 (46.8–55.2)	1701, 50.3 (42.7–57.9)
Girl	2215, 49.0 (44.8–53.2)	1681, 49.7 (42.1–57.3)
*Ethnicity*		
Hispanic	2170, 48.3 (38.8–57.8)	1661, 49.1 (34.7–63.4)
Non-Hispanic	2350, 51.7 (42.2–61.2)	1721, 50.9 (36.6–65.3)
*Parental educational attainment*		
Less than high school	719, 15.9 (10.9–20.9)	480, 14.2 (9.1–19.2)
High school diploma or GED	1049, 23.2 (20.2–26.1)	933, 27.6 (22.1–33.1)
More than high school	2753, 60.9 (54.1–67.7)	1968, 58.2 (48.5–68.0)
Sleep duration *		
Less than 8 h	2201, 48.7 (44.1–53.4)	2401, 71.0 (64.6–77.5)
8 h or more	2391, 51.3 (46.6–55.9)	981, 29.0 (22.5–35.4)
*Daily television (TV) screentime*		
Do not watch TV	334, 7.4 (5.7–9.1)	254, 7.5 (5.0–9.9)
Less than 2 h	1645, 36.4 (33.2–39.7)	1221, 36.1 (33.9–38.3)
2 h or more	2540, 56.1 (51.7–60.6)	1907, 56.4 (53.3–59.6)
Daily video/computer gaming time *		
Do not play video games	1455. 32.2 (27.6–36.9)	1562, 46.2 (41.0–51.4)
Less than 2 h	1604, 35.5 (30.4–40.6)	1004, 29.7 (26.4–33.1)
2 h or more	1460, 32.3 (28.4–36.2)	815, 24.1 (17.9–30.3)

95% CI—95% confidence interval. *—Proportions are significantly different between 8th and 11th graders at *p* ≤ 0.05.

**Table 2:** Weighted logistic regression results for association between electronic media use and short sleep duration in Texas 8th graders.

	OR ^1^	95% CI	*p*-Value
**Boys ^2^** (n = 2237)			
*Daily* *TV screentime*			
Do not watch TV	Ref	--	--
<2 h	0.9	0.4–2.0	0.9
2+ h	0.9	0.4–2.0	0.9
*Daily video/computer gaming time*			
Do not play video games	Ref	--	--
<2 h	2.8	1.4–5.7	0.004
2+ h	3.6	1.6–8.1	0.003
**Hispanic Girls** (n = 1648)			
*Daily TV screentime*			
Do not watch TV	Ref	--	--
<2 h	0.4	0.2–0.7	0.006
2+ h	0.4	0.2–0.8	0.002
*Daily video/computer gaming time*			
Do not play video games	Ref	--	--
<2 h	1.2	0.8–1.9	0.4
2+ h	2.3	1.1–4.7	0.02
**Non-Hispanic Girls** (n = 635)			
*Daily TV screentime*			
Do not watch TV	Ref	--	--
<2 h	2.6	0.9–7.7	0.05
2+ h	2.9	1.0–8.4	0.05
*Daily video/computer gaming time*			
Do not play video games	Ref	--	--
<2 h	1.0	0.6–1.5	0.8
2+ h	1.3	0.6–2.6	0.5

^1^ Odds ratio estimates, adjusted for parental educational attainment. In addition, the boys’ model results were adjusted for ethnicity (Hispanic or non-Hispanic). ^2^ Results for 8th grade boys of all races/ethnicities. Because no significant interaction between ethnicity and either type of electronic media use was observed for boys, we did not stratify 8th grade boys’ results by ethnicity.

**Table 3:** Weighted logistic regression results for association between electronic media use and short sleep duration in Texas 11th graders.

	OR ^1^	95% CI	*p*-Value
**Boys** (n = 1675) ^2^			
*Daily TV screentime*			
Do not watch TV	Ref	--	--
Less than 2 h	0.2	0.1–0.5	0.001
2 h or more	0.2	0.1–0.5	<0.001
*Daily video/computer gaming time*			
Do not play video games	Ref	--	--
Less than 2 h	0.9	0.5–1.8	0.8
2 h or more	1.4	0.7–0.5	0.4
**Girls** (n = 1707) ^2^			
*Daily TV screentime*			
Do not watch TV	Ref	--	--
Less than 2 h	0.5	0.2–1.1	0.09
2 h or more	0.2	0.1–0.6	0.002
*Daily video/computer gaming time*			
Do not play video games	Ref	--	--
Less than 2 h	1.0	0.7–1.3	0.8
2 h or more	0.7	0.3–1.4	0.3

^1^ Odds ratio estimates, adjusted for parental educational attainment and ethnicity (Hispanic or non-Hispanic). ^2^ Models with and without an interaction term for ethnicity resulted in similar estimates of the association between video/computer gaming time and sleep duration. As such, interaction models with ethnicity are not presented for 11th graders.

## Data Availability

Data can be found at https://span-interactive.sph.uth.edu/ (accessed on 12 January 2026).

## Supplementary Materials

The following supporting information can be downloaded at https://www.mdpi.com/article/10.3390/children13010127/s1, Table S1: Weighted logistic regression results for association between electronic media use and short sleep duration for the total sample (N = 7902).

## Abbreviations

The following abbreviations are used in this manuscript: BMI—body mass index; CDC—Centers for Disease Control and Prevention; CI—confidence interval; DAG—directed acyclic graph; DSHS—Department of State Health Services; OR—odds ratio; SPAN—School Physical Activity and Nutrition Survey; TV—television.
