# Supplementary material for: The Differential Associations Between Passive and Interactive Screentime and Sleep Duration Among 8th and 11th Grade Adolescents
Source: Children (Basel). 2026 Jan 15;13(1):127. doi: 10.3390/children13010127 (PMC12840330; doi:10.3390/children13010127)
Supplement: Supplementary file 1 [file children-13-00127-s001.zip › children-4074548-supplementary.pdf]

Supplementary Table S1. Weighted logistic regression results for association between electronic media use and short sleep duration for the total sample (N=7,902).

	OR	95% CI	*P*-value
Daily TV screentime			
Don't watch TV	Ref	--	--
< 2 hours	1.4	1.1-1.7	0.001
2+ hours	1.3	1.1-1.6	0.003
Daily video/computer gaming time			
Don't play video games	Ref	--	--
< 2 hours	0.9	0.8-1.0	0.09
2+ hours	0.7	0.6-0.8	<0.001
Gender			
Female	Ref	--	--
Male	1.4	1.3-1.5	
Grade			
8th Grade	Ref	--	--
11th Grade	0.4	0.3-0.4	<0.001
Parent Education Level			
Less than high school	Ref		
High school/GED	0.9	0.8-1.0	0.21
Some college	0.9	0.8-1.1	0.64
College degree	1.0	0.9-1.2	0.96
Graduate/professional degree	1.0	0.9-1.2	0.86
Ethnicity			
Hispanic	Ref		
Non-Hispanic	1.0	0.9-1.1	0.76
